# Adaptability of Assistive Mobility Devices and the Role of the Internet of Medical Things: Comprehensive Review

**DOI:** 10.2196/29610

**Published:** 2021-11-15

**Authors:** Daniel Ayo Oladele, Elisha Didam Markus, Adnan M Abu-Mahfouz

**Affiliations:** 1 Department of Electrical, Electronic and Computer Engineering Central University of Technology Bloemfontein South Africa; 2 Council for Scientific and Industrial Research Pretoria South Africa

**Keywords:** internet of medical things framework, internet of things, adaptability, multisensor fusion, mobility aids, user system interface, assistive mobility devices, mobile phone

## Abstract

**Background:**

With the projected upsurge in the percentage of people with some form of disability, there has been a significant increase in the need for assistive mobility devices. However, for mobility aids to be effective, such devices should be adapted to the user’s needs. This can be achieved by improving the confidence of the acquired information (interaction between the user, the environment, and the device) following design specifications. Therefore, there is a need for literature review on the adaptability of assistive mobility devices.

**Objective:**

In this study, we aim to review the adaptability of assistive mobility devices and the role of the internet of medical things in terms of the acquired information for assistive mobility devices. We review internet-enabled *assistive mobility* technologies and non–internet of things (IoT) assistive mobility devices. These technologies will provide awareness of the status of adaptive mobility technology and serve as a source and reference regarding information to health care professionals and researchers.

**Methods:**

We performed a literature review search on the following databases of academic references and journals: Google Scholar, ScienceDirect, Institute of Electrical and Electronics Engineers, Springer, and websites of assistive mobility and foundations presenting studies on assistive mobility found through a generic Google search (including the World Health Organization website). The following keywords were used: *assistive mobility OR assistive robots*, *assistive mobility devices*, *internet-enabled assistive mobility technologies*, *IoT Framework OR IoT Architecture AND for Healthcare*, *assisted navigation OR autonomous navigation*, *mobility AND aids OR devices*, *adaptability of assistive technology*, *adaptive mobility devices*, *pattern recognition*, *autonomous navigational systems*, *human-robot interfaces*, *motor rehabilitation devices*, *perception*, and *ambient assisted living*.

**Results:**

We identified 13,286 results (excluding titles that were not relevant to this study). Then, through a narrative review, we selected 189 potential studies (189/13,286, 1.42%) from the existing literature on the adaptability of assistive mobility devices and IoT frameworks for assistive mobility and conducted a critical analysis. Of the 189 potential studies, 82 (43.4%) were selected for analysis after meeting the inclusion criteria. On the basis of the type of technologies presented in the reviewed articles, we proposed a categorization of the adaptability of smart assistive mobility devices in terms of their interaction with the user (user system interface), perception techniques, and communication and sensing frameworks.

**Conclusions:**

We discussed notable limitations of the reviewed literature studies. The findings revealed that an improvement in the adaptation of assistive mobility systems would require a reduction in training time and avoidance of cognitive overload. Furthermore, sensor fusion and classification accuracy are critical for achieving real-world testing requirements. Finally, the trade-off between cost and performance should be considered in the commercialization of these devices.

## Introduction

### The Internet of Things

Internet technology has experienced remarkable progress since its early stages. It has become a vital transmission framework aiming to connect anyone and anything at any time to any service [1]. The basic idea of the internet of things (IoT) is to allow an autonomous and secure connection and exchange of data between real-world devices and app [2]. IoT has become a crucial factor in next-generation technology and the whole business spectrum. It is the seamless interconnection of uniquely identifiable smart objects, sensors, and informatics systems within today’s internet infrastructure with extended benefits. Typically, benefits include the advanced interconnectivity of these devices, systems, and services that go beyond machine-to-machine scenarios [3]. The impact of IoT has led to its application in several fields for enhancing network operation and the user’s quality of experience [1]. These fields include transportation, health care, industrial automation, and public safety management [4].

### Smart Health Care and Assistive Mobility

Health care is an attractive application area for IoT [5]. IoT has the potential to give rise to many medical apps, such as remote control and health monitoring, fitness programs, chronic diseases, and elderly care [3]. For instance, with a monitoring app, the patient can transmit daily or weekly blood pressure readings. This enables their physician to detect a problem and intervene earlier. Smart health care can be referred to as an organic whole of conventional mobile devices used with wearable medical devices, assistive mobility devices, and IoT gadgets (such as implantable or ingestible sensors). This can also be referred to as the internet of medical things (IoMT). This organic whole enables continuous patient monitoring and treatment, even when patients are at their homes. Examples of these assistive mobility devices are pressure monitors, glucometers, smartwatches, smart walkers, smart wheelchairs, smart contact lenses, and way finders [6].

With an increase in the percentage of people with some form of disability [7-10], assistive mobility has become an important aspect of research and has gained a lot of attention from researchers in recent years. Mobility has to do with an individual’s ability to move his or her body within an environment and the ability to manipulate objects. This ability can be hampered by impaired body functions or structures and limit the individual’s functioning, independence, and overall well-being [11]. Assistive mobility is a broad term used to refer to the use of aid (of any kind) to improve the mobility of an impaired individual.

Technology has been a tool used by researchers and companies to address the limitations in mobility caused by some form of impairment. For this reason, literature reviews and surveys have been conducted on assistive technologies for individuals with some form of disability. Although literature reviews have been conducted on specific assistive mobility technologies (such as smart wheelchairs, scooters [12,13], and smart canes [14]), gait rehabilitation devices (such as smart walkers, lower-limb exoskeletons, and smart crutches) [15-19], and how these technologies have addressed mobility limitations of impaired individuals, the review of all elements needed in the adaptability of assistive mobility devices to the user in terms of information used requires more attention.

Related literature review papers have paid attention to specific elements needed in the adaptability of mobility devices, such as the survey of alternative input and feedback methods, including haptic [20], visual, and auditory [21,22] methods, as sensory replacement and sensory augmentation for certain sensory impairments and the survey of computer vision (CV) and machine learning techniques [23,24] for autonomous driving. More closely related surveys [25] approached the categorization of assistive technology based on users’ needs but concentrated on the cross-application of CV for categorization. An older review in 2012 [11] focused on the seamless integration of the capabilities of the user and the assistive technology for mobility. These related reviews highlighted the adaptability of assistive technologies as crucial in the technological advancement of mobility devices. However, we believe that an approach to the adaptability of assistive mobility devices in terms of information used has not been considered.

The objective of this study is to primarily focus on a literature review of the adaptability of assistive mobility devices and the role of IoMT in terms of the acquired information for assistive mobility devices. Internet-enabled assistive mobility technologies and non-IoT assistive mobility devices will be reviewed. The technologies reviewed will provide insight into some important themes and serve as a source and reference for information on adaptive assistive mobility technology to health care professionals and researchers. More specifically, we aim to contribute to the following:

- Identifying the major areas crucial for the adaptability of internet-enabled assistive mobility technologies (such as smart wheelchairs, smart walkers, smart canes, and scooters) and other non-IoT assistive mobility devices (such as regular walkers, wheelchairs, canes, crutches, walkers, orthoses, and prostheses) to its intended users
- Categorization of the adaptability of assistive mobility devices in terms of the acquired information into three major areas: user system interfaces (USIs), perception and sensor fusion techniques, and IoMT frameworks
- Highlighting the role that IoMT plays in the adaptability of assistive mobility devices

## Methods

We selected a list of studies and references to review the adaptability of assistive mobility devices and IoT frameworks for assistive mobility to be included in the literature search. The data sources used to search for the items to be included in this review were the following databases of academic references: Google Scholar (including ResearchGate), ScienceDirect, Institute of Electrical and Electronics Engineers, Springer, and websites of assistive mobility and foundations presenting studies on assistive mobility found through a generic Google search (including the World Health Organization website).

The search criteria included the following keywords and combinations thereof: *assistive mobility OR assistive robots*, *assistive mobility devices*, *internet-enabled assistive mobility technologies*, *IoT Framework OR IoT Architecture AND for Healthcare*, *assisted navigation OR autonomous navigation*, *mobility AND aids OR devices*, *adaptability of assistive technology*, *adaptive mobility devices*, *pattern recognition*, *autonomous navigational systems*, *human-robot interfaces*, *motor rehabilitation devices*, *perception*, and *ambient assisted living*.

As these combinations of data sources and keywords returned a vast number of results, we selected the following inclusion criteria to identify the most relevant sources: (1) language should be English, (2) date range should be in the past 12 years (2008-2020)—most articles were published within the past 5 years to reflect the state-of-the-art (since 2015), and older references were made to technologies that substantially shaped the future direction of assistive mobility devices—and (3) its relevance should be in internet-enabled assistive mobility technologies or non-IoT assistive mobility devices.

The PRISMA (Preferred Reporting Items for Systematic Reviews and Meta-Analyses) criteria were applied [26]. The screening of titles and abstracts was performed by DAO and EDM and reviewed by DAO, EDM, and AMAM. Full texts were reviewed in a second screening.

## Results

### Overview

After excluding results with titles that were not relevant to this study, the literature search identified 13,286 abstracts, of which 189 (1.42%) potential studies were selected for a detailed full text review.

We used the following exclusion criteria to identify the most relevant sources and reduce the number of literature search results: (1) no relevance to internet-enabled assistive mobility technologies or non-IoT assistive mobility devices in terms of the acquired information, (2) full text not available, (3) no report on promises for user adaptability as a result of simulation testing or using the technology, (4) no description of the technology, and (5) no additional contribution to the review findings compared with the previously reviewed articles.

**Figure 1:**
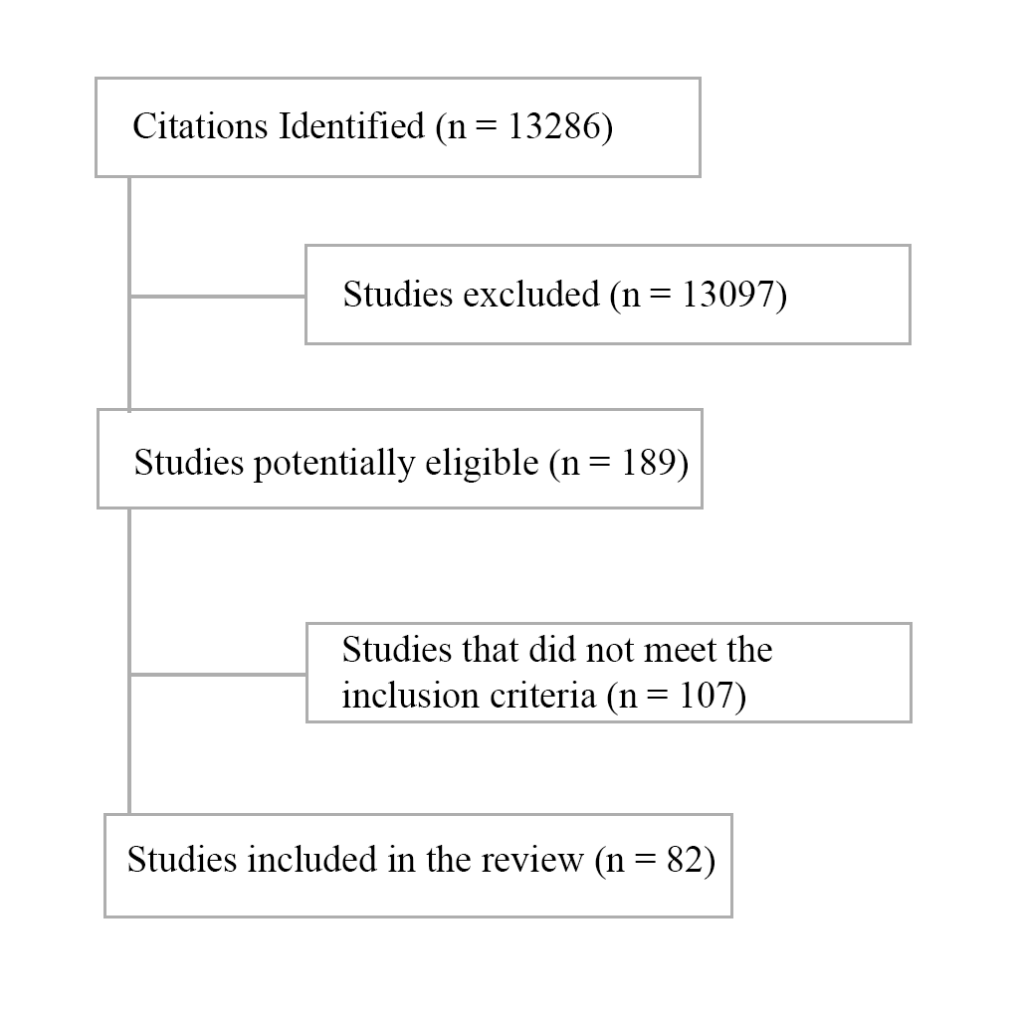
Flow diagram of search results. IoT: internet of things.

Of the 189 potential studies, 82 (43.4%) studies remained for analysis after meeting the inclusion criteria. Some studies contributed to more than one section in this review (Figure 1).

To perform a literature review based on the type of technologies presented in the reviewed articles, we proposed a categorization of the adaptability of smart assistive mobility devices in terms of their interaction with the user (USI), perception techniques, and communication and sensing frameworks.

In recent years, advances in technology have helped to improve the quality and efficiency of assistive mobility devices. The use of traditional assistive mobility devices by users with some form of cognitive, sensory, or intellectual impairment requires the help of medical personnel or a caregiver for navigation assistance with difficult daily maneuvering tasks. To accommodate users who find operating standard mobility devices difficult or impossible, several researchers have used technologies originally developed for mobile robots to create smart mobility devices [27], such as smart wheelchairs and smart ambulatory devices. These assistive mobility devices are made smart by attaching computers, actuators, and sensor subsystems to the traditional assistive mobility device to provide easy maneuvering, system localization, object detection, and other sensory, cognitive, and health monitoring functions [12,28-30].

### USI (Input and Output Methods)

#### Overview

With the advent of smart assistive mobility devices, some assistive devices have become too complex to use. In addition, improper characteristics of the target users have resulted in numerous assistive mobility projects failing to transition to real-world use [31]. For this reason, the adaptability of assistive mobility devices is very important. Some users of assistive mobility devices have comorbidities, such as sensory impairment for users with spinal cord injury (SCI) or mental health challenges because of aging or depression. This impairment needs to be taken into account in the design of efficient assistive mobility devices. Assistive mobility devices should be designed to continually evaluate and correct their actions based on their perception of the needs of the user [32]. Mobility impairment of patients can be largely classified into 2 functional groups. The first group includes individuals with a total loss of ability to move by themselves and with a high risk of confinement in bed, and, consequently, they suffer the effects of prolonged immobility. Examples are patients with complete SCI, advanced neurodegenerative pathologies, severe lower-limb osteoarthritis, and fractures of the spine or lower-limb bones. The suitable kind of assistive mobility technology for this group is called the alternative device. Examples are wheelchairs and autonomous vehicles (AVs). The second group includes individuals with partial loss of mobility, presenting different levels of residual motor capacity that can be powered by assistive mobility devices. The suitable type of assistive device for this group of individuals is the augmentation (rehabilitation) device. Examples are wearable orthoses and prostheses or external devices (such as canes, crutches, and walkers) [28,33]. Notwithstanding the functional group of mobility-impaired patients, USIs are a crucial element in the adaptability of assistive mobility devices. USI has to do with the acquisition of information from the user, the interpretation of this set of acquired information, and the available feedback methods that can be understood by its intended users.

USIs for assistive mobility devices are categorized based on the type of sensors and actuators used for acquisition of user’s information. These includes CV, brain-computer interface (BCI), and voice, touch, and haptic feedback [12]. The USI technologies presented below are categorized as follows: BCI, CV interface (CVI), and auditory and haptic interface.

#### BCI System

BCI generally refers to a system that measures and uses signals produced by the central nervous system. This interface enables useful functions for people with disabilities caused by neuromuscular disorders such as amyotrophic lateral sclerosis, cerebral palsy, stroke, or SCI [34]. The basic components of the BCI are signal acquisition, signal processing, and the effector or output device [35]. Signal acquisition can be invasive or noninvasive [35]. Over the past decade, many educative literature reviews and surveys have been conducted and documented by researchers on the definition, mode of operation, classifications, functionality, and applications of BCI [34-37].

The adaptability of assistive mobility devices for users with neuromuscular disorders has led to the adoption of BCI as a suitable means of user-machine communication for simple mobility tasks. BCI offers limited navigation control capabilities to assistive mobility devices. To improve the navigational abilities offered by BCI, models proposed by researchers integrate BCI with other USI and machine learning tools. For example, Rebsamen et al [38] and Long et al [39] proposed a P300-based BCI wheelchair for the execution of commands for a set of predefined locations. Some auxiliary sensors were also integrated for collision avoidance during navigation. Long et al [39] proposed a hybrid BCI system comprising a motor imagery (MI)-based mu rhythm and the P300 potential. This model was designed for the directional and speed control of a brain-actuated simulated wheelchair or a real wheelchair. Kim et al [40] proposed a prototype that addressed a user’s loss of vision in their environment. The prototype uses the steady-state somatosensory evoked potential (SSSEP) paradigm to control a wheelchair by using specific frequencies and vibrations of different body parts to elicit brain responses. They also recommended the use of an auxiliary autonomous navigation system to improve performance. An asynchronous MI-based BCI protocol system control was proposed by Carlson and del R Millan [41] to improve navigational control with the help of 10 lost-range sonar sensors and 2 webcam cameras.

Furthermore, a teleoperation control for a robotic exoskeleton system based on the steady-state visual evoked potentials (SSVEPs) BCI and visual feedback was proposed by Qiu et al [42]. A camera was used to capture video for visual feedback, and a local adaptive fuzzy controller was used to drive the exoskeleton to track the intended trajectories in the human operator’s mind. The controller was also used to provide, in a convenient way, dynamic compensation with minimal knowledge of the dynamic parameters of the exoskeleton robot.

#### Auditory and Haptic Interface

Individuals with mobility impairments having visual, hearing, or tactile disabilities require the use of an alternative sensory ability for effective communication with assistive mobility devices. Auditory interfaces are designed to take advantage of hearing ability as a substitute for visual or tactile impairment. On the other hand, haptic interfaces are designed to take advantage of the users’ tactile ability as a substitute for visual, auditory, or motor impairment [43]. An extensive review has been conducted on haptic assistive technology as a means of communication for individuals with some form of sensory impairment, such as visually and auditorily impaired individuals [20,21,31,43]. Parker et al [22] also reviewed the positive effect of visual and auditory feedback on motor skills of poststroke patients during gait rehabilitation. This subtopic presents recent auditory and haptic interface technologies for individuals with mobility impairments.

Haptic technology has been a beneficial USI for certain impaired users. It has found its application in many areas for the monitoring of users’ progress and for navigational assistance. It has been successfully implemented in the design of exoskeletons (such as orthoses and wearable devices for grasping and assisted movement), smart walkers, smart crutches, and smart wheelchairs. Like haptic technology, auditory technology is also used as an alternative navigational control for individuals with mobility impairment and as a navigational guide or feedback for patients with visual impairments. Many researchers have integrated haptic or auditory technology for navigational control, navigation assistance, or feedback of assistive mobility devices. Wearable devices such as the Jet Propulsion Laboratory BioSleeve [44,45], the wireless tongue drive system (TDS) to smartphone (iPhone) electric powered wheelchair (PWC; TDS to smartphone (iPhone) electric-PWC [TDS-iPhone-PWC]; [46]), and the MyoSuit [47] were designed using haptic technology for navigational control and aided mobility, respectively. The JPL BioSleeve is a wearable, hands-free gesture recognition interface that decodes as many as 20 discrete hand and finger gestures and can estimate the continuous pose of the arm. It was designed using surface electromyography (EMG) sensors, an inertial measurement unit (IMU), and embedded software. EMG and IMU acquire gesture and pose signals, whereas the embedded software classifies the signals and maps the result to commands. The wireless TDS-iPhone-PWC uses a TDS comprising a wearable TDS headset, a magnetic tongue barbell, a control unit, and magnetic sensors. The prototype wirelessly sends up to six distinct control commands to an iPhone for the navigation of a PWC after calibration training using a PC. MyoSuit is a lightweight, lower-limb, soft, wearable robot (exoskeleton) for rehabilitation training that allows active contributions from users in residual mobility. It was designed to estimate interlimb angles and trunk postures using a five-segment body model acquired from IMU. It also determines which model is suitable for the user.

Other examples of haptic-based technology for adaptive mobility include the smart cane [48], intelligent control smart walker [49], and learning shared control of an assistive robotic transport for adults wheelchair-powered platform [50]. The smart cane was designed using a force sensor for the measurement of the exerted weight and IMU for pose estimation. The intelligent control smart walker was designed to use a force sensor to control acceleration. The learning shared control of an assistive robotic transport for adults wheelchair-powered platform was designed to regulate the level of assistance between the user and the robot by matching the location and amount of offered assistance on different trajectories.

Some recent technologies integrate multiple technologies for USI in the process of adapting mobility devices to a desired group of disabilities. An example is the electronic mobility cane (EMC) [51], which was designed using multiple sensors to contract the logical map of the surrounding environment and give feedback of the priority information to the user without causing any information overload. Another example is the EyeCane [52], which is an electronic travel aid or electronic travel support that aims to increase the perception of the environment using multiple sensors for distance estimation, navigation, obstacle detection, and feedback to the user. The last example is the multiple controlled interfaces smart wheelchair [53], which was designed to accommodate a variety of impaired individuals. It is a prototype wheelchair with multiple control options (voice, gesture, and joystick input). Another recently explored area is CV to sound technology, which is further discussed in the following section.

#### CVI System

As humans, we perceive 3D structures of the world around us with apparent ease [54]. The ability of computers to *see* and understand the world just like humans do gave birth to the research of CV. CV is a field of study that seeks to develop mathematical techniques that enable computers to interpret and understand the visual world (images and videos) accurately in the same way as humans do. CV starts with the acquisition of data or capturing of information, which is done with the help of vision and depth (3D ranging) sensors, such as image-based sensors (mono and stereo or depth cameras), laser-based depth sensors (light detection and ranging, laser scanner, and infrared light), sound-based depth sensors (sound navigation and ranging and ultrasonic), and radio detection and ranging sensor [55].

There are many applications of CV [56-60], and one such application is CV USI for adaptive assistive mobility devices. An example is the visual servoing-controlled wheelchair proposed by Pasteau et al [61]. The proposed smart wheelchair uses 3 cameras for autonomous corridor following and doorway passing. Another example is the autonomous scooter navigational system proposed by Mulky et al [13] to assist people with independent transportation challenges and recognition of the fine-grained world around them. This was achieved using a long-range eye-safe laser (up to 60 m) and a stereo vision camera. Finally, the user-adaptive control intelligent walker proposed by Chalvatzaki et al [62] used CVI technology (laser range finder) to estimate the human state and classify a patient’s mobility status.

CVI mostly integrates haptic or auditory technologies for user feedback and finds its applicability in user or environmental perception for assistive control, monitoring, and sensory substitution devices (SSDs). For instance, the sound of vision SSD technology [63-65] assists people with visual impairment with navigation by converting visual perception to (spatial) sound or haptic feedback. Usually, sound of vision SSDs comprise data acquisition operational modes, an image processing pipeline, and a feedback system [63-65]. An example of a multimodal USI is the iChair, a multimodal input platform that accepts commands from voice, touch, proximity switch, and head-tracking cameras and provides seamless access and control for users with severe disabilities [30].

CVI is the first phase toward autonomous navigation and is a crucial part of perception and multisensor fusion techniques [24].

### Perception for Adaptability (Autonomous Navigation)

Autonomous navigation simply refers to the ability of a robot or vehicle to sense its environment and navigate accurately without human input or assistance [66]. AVs or autonomous robots (ARs) are meant to be intelligent enough to perceive, predict, decide, plan, and execute their decisions in the real world [24]. The main difference between AVs and ARs is in the fact that AVs address road networks where traffic rules have to be obeyed, whereas ARs have to cope with open environments without many specific rules to follow only to reach the final destination [67]. There are six different levels of driving autonomy (Table 1), as published by the Society of Automotive Engineers International in 2021, ranging from no automation at level 0 to full automation at level 5 [66,68]. Following the Society of Automotive Engineers International definition, existing AVs and ARs in 2021 are not fully autonomous. Mobility aids can be seen as a type of AR, and the adaptability of the mobility aid is dependent on its ability to make intelligent navigational decisions with limited to no intervention by its user.

**Table 1:** Summary of the Society of Automotive Engineers (SAE) automation levels.

SAE [66,68] levels	DDT^a^		Driving supervision (DDT fallback)	Scenarios (ODD^b^)
	Vehicle controls	Environment monitoring (OEDR^c^)		
0: no driver automation	Driver	Driver	Driver	N/A^d^
1: driver assistant	Driver	Driver	Driver	Limited
2: partial driving automation	Driver and vehicle	Driver	Driver	Limited
3: conditional driving automation	Vehicle	Vehicle	Driver and vehicle	Limited
4: high driving automation	Vehicle	Vehicle	Vehicle	Limited
5: full driving automation	Vehicle	Vehicle	Vehicle	Unlimited

^a^DDT: dynamic driving task.

^b^ODD: operational design domain.

^c^OEDR: object and even detection and response.

^d^N/A: not applicable.

**Figure 2:**
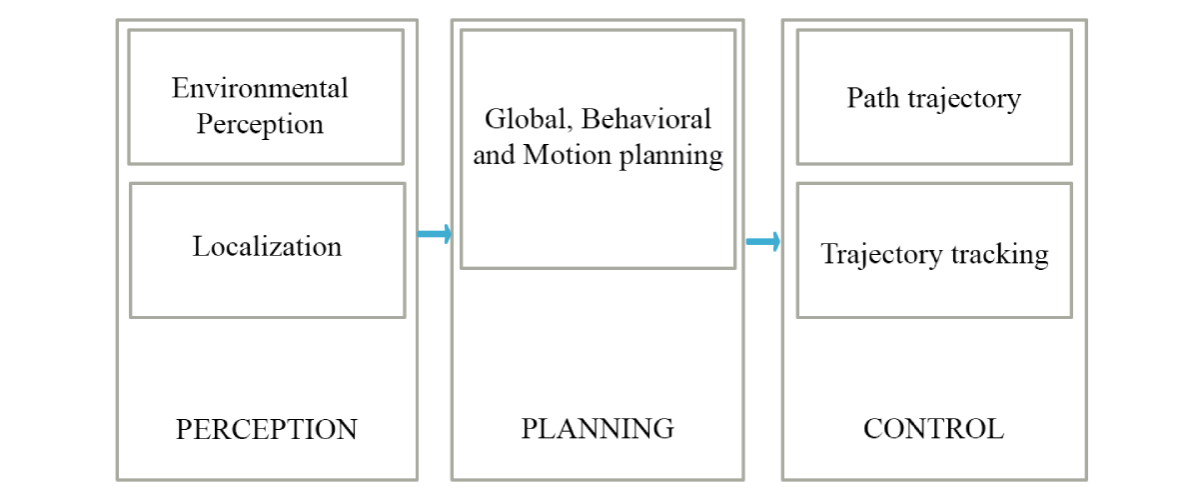
Summary of an autonomous system.

Generally, there are three main steps in the operation of an autonomous system (Figure 2): the perception stage (environmental perception and localization), the path planning stage, and the control stage. The perception stage, which is the first stage of a self-driving system, is a crucial aspect of autonomous navigation or self-driving robots. The perception stage majorly comprises environmental perception and localization [69]. The success of perception is largely dependent on the accuracy of the sensors used in the data acquisition. A combination of sensors helps improve accuracy and confidence for the best decision task in environmental perception and autonomous navigation. Although there are high-accuracy sensors that can work alone without exhibiting some of the limitations common to regular sensors, they are often unavailable because of their operating limits and high costs. This makes them impractical for use in real-world applications [70]. This limitation, which is common to regular sensors, has led to the need for multisensor fusion to improve accuracy and confidence. Multisensor fusion has to do with the process of combining information from different sensors to provide a robust and complete description of the environment or process of interest [71]. Detailed literature about each stage has been reviewed [67,69,72,73].

Many recent assistive mobility technologies have made advancements in striving toward fully autonomous navigation, such as the technologies discussed in the *USI (Input and Output Methods)* section (Tables 2-4). Some examples include the P300 BCI-based controlled wheelchairs [38,74], as shown in Table 2. The authors designed the prototype to achieve a level of autonomy using cheap sensors. A bar code was used for global positioning, and a proximity sensor was used for collision avoidance. The use of multisensor fusion was not adopted in this prototype. Feature extraction classifiers (stepwise linear discriminant analysis and support vector machine [SVM]) were used to adequately process the BCI information needed to autonomously navigate the wheelchair from the point of command to its predefined location without the help of its user.

In Table 3, the multicontrolled wheelchair [53] used an algorithm for the control and execution of commands to check for predefined commands and execute them in the navigation and speed control of the wheelchair. An ultrasonic sensor was used for autonomous navigation.

The visual servoing-controlled wheelchair [61], as shown in Table 4, used CV with the classic Gaussian sphere projection framework and line segmentation algorithm for corridor following. A door detection and tracking framework (for indoor navigation tasks) and a 2D edge tracker was inspired by the moving edges algorithm for autonomous doorway passing. In addition, the autonomous scooter navigation [13], as shown in Table 4, used CV and the graph-based simultaneous localization and mapping algorithm for steering control and autonomous navigation.

**Table 2:** Brain-computer interface (BCI) technologies for adaptive assistive mobility devices.

Brain signals and auxiliary sensors	Classifier for feature extraction	Output command	Contributions	Drawbacks
P300 (laser scanner) [74]	Stepwise linear discriminant analysis	A predefined set of locations and stops	High accuracy, no training required, and autonomous navigation after successful selection	Low information transfer rate, predefined paths, limited testing scenarios, and possible fatigue after long focus period of the eye on the target stimulus
P300 (odometer, barcode scanner, and a proximity sensor) [38]	Support vector machine	A predefined set of locations and stops	Same as Rebsamen et al [38]	Same as Rebsamen et al [38] and a modified environment requires an update of the guiding path
MI^a^-based mu rhythm and the P300 [39]	One versus the rest common spatial patterns transformation matrix	Left, right, accelerate, and decelerate	Improved performance	Limited testing scenarios and possible fatigue after long focus period of the eye on the target stimulus
MI-based BCI (10 sonar sensors and 2 webcams) [41]	Gaussian classifier	Left, right, and keep moving forward	Spontaneous and shared control	Limited testing scenarios, requires extensive training, and limited classes (typically three)
Steady-state visual evoked potentials (camera and adaptive fuzzy controller) [42]	Frequency recognition algorithm based on multivariable synchronization index	Left, right, upwards, and downwards	Teleoperation control of an exoskeleton using a brain-machine interface	Possible fatigue after a long focus period of the eye on a target stimulus and a significant reduction in recognition accuracy for inexperienced subjects
Steady-state somatosensory evoked potential [40]	Regularized linear discriminant analysis	Turn left, turn right, and move forward	Spontaneous, first of its kind, and addressed the possible fatigue after a long focus period of the eye on the target stimulus	Only healthy subjects were used, with limited testing scenarios (two)

^a^MI: motor imagery.

**Table 3:** Brain-computer interface technologies for adaptive assistive mobility devices.

Technology name (type): additional sensors	Machine learning tools	Contributions	Drawbacks
TDS-iPhone-PWC^a^ (haptic): magnetic sensors [46]	Sensor signal processing algorithm	An alternative USI^b^ for people with spinal cord injury or upper limb paralysis	Tongue piercing can be a painful and uncomfortable option for some users. Extensive training is required for calibration.
Intelligent smart walker (haptic): force or torque sensor [49]	N/A^c^	An intuitive rule-based speed controller for a smart walker	Young and healthy subjects were used, so the result is not a true representation of the typical users of the walker.
EyeCane (CVI^d^, haptic, and auditory): infrared emitters, auditory frequency actuator, and tactile actuator [52]	N/A	Low cost, lightweight, small and easy to use electronic travel aid for distance estimation and navigational assistance, long battery life (one whole day), intuitive to the user, and short training time (<5 minutes)	Only an indoor experiment was conducted.
Electronic mobility cane (CVI, haptic, and auditory): liquid detection, 6 ultrasonic sensors, a metal detector, a microvibration motor, and a mono earphone [51]	A novel algorithm named *way-finding with reduced information overload.*	Offers real time multiple obstacle detection and way-finding assistance simultaneously to patients with visual impairments by an auditory (voice message) and tactile (vibration) feedback	Extensive training time (20 hours); the cognitive and perceptual load has not been ascertained
Jet Propulsion Laboratory BioSleeve (haptic): electromyography and IMU^e^ sensors [44,45]	A multiclass support vector machine classifier	Intuitive control of robotic platforms by decoding as many as 20 discrete hand and finger gestures	Has not yet been integrated and tested with assistive mobility aids to determine its applicability
Smart cane (haptic): IMU and FSR^f^ sensors [48]	C4.5 decision tree, artificial neural network, support vector machine, and naive bayes	To monitor and distinguish between different walk-related activities during gait rehabilitation	Fall and near-fall detection was not considered in its design and implementation.
An ARTA^g^ power wheelchair platform (CVI and haptic): haptic controller, laser scanner, SICK laser measurement, and IMU sensor. [50]	Gaussian process regression model	Implementation of a learned shared control policy from human-to-human interaction	The efficiency of the learning process is dependent on the human assistant, who is prone to errors and might miss out on the certain intent of the user.
Multiple controlled interfaces smart wheelchair (haptic and auditory): microphone, joystick, leap motion, and ultrasonic sensor [53]	An algorithm for the control and execution of commands	Multiple control interfaces	Lack of details on the performance of each interface and limited testing scenarios
MyoSuit (haptic): IMU sensor and two electric motors [47]	N/A	Lightweight, soft wearable robot to aid users with a level of residual mobility during locomotion tasks	Only one incomplete spinal cord injury participant was selected for testing, so it is difficult to validate its performance.

^a^TDS-iPhone-PWC: tongue drive system to iPhone electric-powered wheelchair

^b^USI: user system interface.

^c^N/A: not applicable.

^d^CVI: computer vision interface.

^e^IMU: inertial measurement unit.

^f^FSR: force sensitive resistor.

^g^ARTA: assistive robotic transport for adults.

**Table 4:** Computer vision (CV) interface technologies for adaptive assistive mobility device.

Technology name (type): additional sensors	Machine learning tools	Contributions	Drawbacks
See ColOr (CV, auditory and haptic): 3D Kinect, iPad, and Bone-Phones [63]	Multilayer artificial neural network for *object classification,* Kalman filter for *tracking objects* (finger), and randomized forest algorithm for *object detection*	A framework for the coupling of optical sensors in the context of range and color image registration and the development of a sonic code that maps colors and depth into musical instruments	Extensive training was required, and testing was limited to certain scenarios.
Wearable mobility aid for patients with visual impairments (visual, auditory, and haptic): RGBD^a^, vibrotactile glove, and bone-conductive headsets [64]	Stereo vision algorithm and semiglobal matching algorithm; detection: random sample consensus algorithm and Kalman filter; categorization: convolution neural network	Improves on a preliminary prototype of Mattoccia [75], enabling dynamic autonomous mobility capability combining features of electronic travel support and self-localization support in a compact and lightweight setup	Patient feedback from the Mattoccia [75] study was considered, and the result that covered collision rate and cognitive and perceptual overload on tested subjects was not presented.
Visual servoing-controlled wheelchair (vision): 1 camera for corridor following and 2 cameras for ADP^b^ [61]	Classic Gaussian sphere projection framework, door detection and tracking framework, and a 2D edge tracker inspired by the moving edge algorithm	Addresses, in a secure way, the autonomous stability of the wheelchair’s position along corridors and also detects and passes through doorways using visual data	Human input in the control was not considered.
iChair (vision, auditory and haptic): high-definition camera, 3D scanner, 10 LEDs^c^, touch screen and voice recognition app, and head mouse [30]	Light communication algorithm, collision avoidance algorithm, and an emergency and stress detection algorithm	A multimodal input smart wheelchair to identify and classify objects, build 3D maps, and eventually facilitate autonomous navigation	A bug-free human trial has not yet been documented.
CV for patients with visual impairment (vision, auditory, and haptic): A stereo RGB^d^ camera (SC), a depth-of-field camera, and an IMU^e^ [65]	Detection and tracking algorithm, support vector machine classifier, and a class-specific extremal regions for text detection	Addresses the pervasiveness requirement as well as offers sensory substitution via sound feedback to patients with visual impairment	The outdoor performance noted clustering of several objects into a single one and error in identifying lower parts of the object; no outdoor and usability test was documented.
Autonomous scooter navigation (vision): MPU-9250 IMU, long-range laser, and stereo vision camera [13]	A graph-based simultaneous localization and mapping algorithm	Cost-effective and addresses the navigational and localization challenges in an unknown environment by a new hybrid far-field and near-field mapping solution	Extensive documentation of human testing has not been documented.
User-adaptive intelligent robotic walker (vision): laser range finder [62]	Interacting multiple model particle filters with probabilistic data association framework, Viterbi algorithm (human gait estimation), support vector machine classifier, and unscented Kalman filter algorithm	Human state estimation, pathological gait parametrization, and characterization for classifying users associated with risk fall	A test to evaluate the performance of the control strategy with the robotic mobility assistive device and patients was not documented.

^a^RGBD: red green blue and depth.

^b^ADP: autonomous doorway passing.

^c^LED: light emitting diode.

^d^RGB: red green blue.

^e^IMU: inertial measurement unit.

### IoMT Frameworks: Impact of IoMT on the Adaptability of Assistive Mobility Devices

IoMT generally contributes to the adaptability of assistive mobility aids in the monitoring and control by users, caregivers, and medical personnel. The adaptability of assistive mobility devices involves the acquisition of information and the making of intelligent decisions based on the acquired information. This information is obtained from the environment and user via a means of communication (usually an interface). USIs can send and receive information from the user (individuals with some form of disability) to the mobility aid via a communication channel that could be wired or wireless, such as the JPL BioSleeve [44,45] and the TDS-iPhone-PWC interface (Table 3) [46] that can wirelessly control a mobility aid, the P300-based BCI (Table 2) [74] that controls a wheelchair via a wired USB channel, and the autonomous scooter navigation mobility aid [13] that connects its computing module to its hard unit via a wired USB medium or a wireless Bluetooth medium. With the help of IoMT, interconnectivity between mobile devices and their environment and the storage or retrieval of relevant information for control, better autonomy, and monitoring are possible. Many recent surveys and reviews have been conducted on IoMT’s recent technologies, applications, challenges, and opportunities [3,76-78].

In recent years, many researchers have proposed IoMT frameworks for assistive devices that leverage or build on existing IoMT architectures and communication protocols and restructure them (using algorithms or management systems) to suit assistive technologies. For instance, Bae et al [79] proposed a network-based rehabilitation system, for mobility aids (knee assistive devices), as shown in Table 5. The prototype framework distributes the control of the mobility device between the patient’s side and the physiotherapist’s side over a wireless network using the transmission control protocol for internet communication. A modified linear quadratic Gaussian algorithm was used to compensate for packet losses in the wireless network by modeling the losses as Bernoulli random variables. However, only simulations and experiments have been conducted. Therefore, its efficiency in tackling packet loss and robustness against modeling uncertainties, such as interactions with human emotions, has not been evaluated in real-world scenarios.

**Table 5:** Internet of medical things technologies for adaptive assistive mobility devices.

Name of framework	Management system or algorithms	Contributions and functions	Drawbacks
NBR^a^ system framework [79]	Modified linear quadratic Gaussian algorithm	Distributes the control of a mobility device between the patient’s side and the physiotherapist’s side; brings convenience to patients and therapists	Only simulations and experiments have been conducted.
Global concept SEES^b^ framework [64]	Intelligent transportation system	Designed to address the walking and orientation problem*;* functions: user tracking, sending of emergency error or alert messages to patients with visual impairment, obstacle detection, walked distance estimation, surface roughness estimation, and traffic light detection	Only one simple experiment has been conducted.
SHS^c^ framework [80]	Hybrid sensing network, the IoT^d^ smart gateway, and the user interfaces for data visualization and management	Monitoring and tracking of patients, personnel, and biomedical devices in real time; collecting both environmental conditions and patient’s physiological parameters and delivering them to a control center	Use-case scenario testing has not been conducted except for fall detection of 1 patient.
ROS^e^ framework [81]	Navigation, localization, and pick and place algorithm	For the cooperation among SWC^f^ and RW^g^; for the user to be able to interact with and control the SWC as well as any object connected to the RW	At present, the whole architecture has been tested in simulation only.

^a^NBR: network-based rehabilitation system.

^b^SEES: Smart Environment Explorer Stick.

^c^SHS: smart health care system.

^d^IoT: internet of things.

^e^ROS: robotic operating system.

^f^SWC: smart wheelchairs.

^g^RW: robotic workstations.

Yusro et al [82] proposed the global concept Smart Environment Explorer Stick framework that enhances the white cane to assist the navigation of patients with visual impairment. As shown in Table 5, it was designed to address the walking and orientation problem by assisting some of the walking and orientation functions and adopting an active multisensor (ultrasonic, camera, accelerometer, wheel encoder, compass, tactile point-wise, and audio feedback) context-awareness concept. Cellular IPv6 over low-power personal area network communication protocols and routing protocols for low-power and lossy networks were used to help patients with visual impairment to move safely and easily in any environment (indoor and outdoor). However, only one simple experiment was performed. An IoT-aware architecture for smart health care systems (SHSs), applicable to the adaptability of assistive mobility devices, was proposed by Catarinucci et al [80] (Table 5). It promised to guarantee innovative services for the automatic monitoring and tracking of patients, personnel, and biomedical devices within hospitals and nursing institutes in real time. The SHS framework [81] relies on different but complementary technologies, specifically radio frequency identification, wireless sensor networks, and smart mobile, interoperating with each other through a constrained application protocol or IPv6 over low-power personal area network or representational state transfer network infrastructure (Table 5). However, the SHS framework was proposed to demonstrate its feasibility, and it needs to be tested in various use-case scenarios to evaluate its performance. Furthermore, Foresi et al [81] proposed a robotic operating system framework that connects robotic workstations with a smart wheelchair via a Wi-Fi protocol. It was designed to improve the intelligent navigation of the wheelchair and enable interaction between the wheelchair, its user, and any object connected to the robotic workstation. However, only a simulation has been performed on the whole architecture, and a detailed evaluation of its performance is not available.

Although IoMT assistive mobility device frameworks show promising signs to improve the adaptability of mobility aids, most proposed frameworks have not been tested. This is extremely important for evaluating their performance and applicability in adapting mobility aids to their intended users. Notable drawbacks common to IoMT frameworks, such as packet loss, user privacy and security, network robustness and scalability, and commercialization cost [1,83,84], need to be extensively evaluated.

## Discussion

### User System Interaction (Input and Output Methods)

#### BCI Systems

BCIs can generally be categorized into four types: P300, SSVEP, event-related synchronization or desynchronization, and SSSEP. P300 is an endogenous response to an oddball stimulus. A positive wave is evoked in response to an event-related potential at a latency of 300 ms (P300). SSVEP is also an endogenous response and is a resonance phenomenon visually evoked by a stimulus modulated at a specific frequency in the brain signals. It occurs in response to the observation of a persistent oscillating visual stimulus. Unlike P300 and SSVEP, event-related synchronization or desynchronization is spontaneously induced by performing mental tasks, such as MI, mental arithmetic, or mental orientation. The SSSEP paradigm is evoked, is endogenous, and spontaneous. The signal is generated in response to the feeling of touch or pressure [35,40,85].

Because of its high accuracy and the need for little to no training, P300 was used by Iturrate et al [74] and Rebsamen et al [38] for the BCI system in the design of the automated navigational wheelchair. Both prototypes still had the drawbacks common with the P300 BCI, such as low information transfer (successful orders per minute), the need for multiple trials for improved accuracy, and the *fatigue experience* that could occur as a result of the long focus period of the eye on the target stimulus. Other drawbacks included the limited testing scenarios conducted on both systems and the fact that only predefined locations could be reached. Rebsamen et al [38] used the path-following mode of operation [12] for automated navigation; therefore, a modification of the environment would require an update to the guiding path. Both prototypes had a limited number of testing scenarios and were carried out on healthy (5) subjects. Long et al [39] adopted the hybrid BCI approach for the control of wheelchair direction and speed using P300 and MI. Emphasis was given to the importance of speed and the use of hybrid BCIs to improve performance and increase command options. Although accuracy was improved (classification performance) and speed control was achieved, testing was limited to only two scenarios (5 subjects for the first and 2 for the second). In addition, the fatigue experience that could occur as a result of the long focus period of the eye on the target stimulus was not addressed.

In an attempt to address the lack of spontaneity associated with P300 and SSVEP, Carlson and del R Millan [41] adopted an MI-based BCI to control a wheelchair. The prototype focused on shared control between the user and the wheelchair, that is, the ability of the wheelchair to take actions (autonomously navigate) concerning the user’s input and its perceived surroundings (using CV). Drawbacks associated with MI BCI, such as limited classes (typically 3 to avoid difficulties in discriminating MI patterns), extensive training time (a few weeks to months) and the calibration time were still evident. It took a much longer time (>160 seconds) for the 2 inexperienced MI BCI patients out of the 4 to complete the task. In addition, if shared control is not properly matched with the user, it could lead to degradation or loss of function and efficiency. Qiu et al [42] attempted to address the complex dynamic uncertainty and input saturation (leading to tracking error), which is common to exoskeleton robots, by using vision compressive sensing, an SSVEP-based BCI (as a reference command), and an adaptive fuzzy controller for control. Limited testing was performed with 2 veterans and 1 greenhorn patient, and the results showed that training was required. Experienced subjects had a significantly better recognition accuracy (approximately 14% difference). To combat the possible fatigue problem and loss of vision to the environment because of the long focus period of the eye on a particular target stimulus, Kim et al [40] adopted the use of SSSEP BCI in the control of a wheelchair. According to Kim et al [40], this prototype is the first of its kind. Although it tested significantly better than its MI BCI–controlled equivalent, tests were limited to only healthy subjects (12) and were conducted mostly by experienced brain-machine interface subjects. In addition, only two testing scenarios were considered.

#### Auditory and Haptic Interface

Although many advances (in USI) have been made in an attempt to factor in individuals with varying disabilities, the extensive evaluation of the efficiency and applicability of these technologies requires more attention. Affordability, accurate detection of environmental sounds, avoidance of cognitive overload of the users, ease of use, weight of devices, and commercialization are important factors to be considered [15,20,21,31,43]. For instance, JPL BioSleeve [44,45], a very promising interface for decoding a large number of gestures (dynamic and static hand positions) at high accuracy, integrates IMU signals with EMG for gesture recognition. Its intended goal of gesture recognition with high accuracy was achieved. However, it is still unclear for which category of users and devices it would be most suitable. Therefore, proper integration and testing need to be performed with existing mobility aids to determine their applicability. TDS-iPhone-PWC [46] was designed to be an alternative USI for people with SCI or upper limb paralysis. Latched, unlatched, and semiproportion control strategies were used to send commands to the wheelchair. The commands included forward, backward, left, and right motions, as well as adjustable speed levels. The results showed that it could effectively be used to both access a computer and drive a power wheelchair in a unified, wireless, unobtrusive, and wearable form. However, tongue piercings can be a painful process, and some patients would be uncomfortable or find it difficult to use this option for control. In addition, results showed that extensive training was required for proper calibration and improved performance (task time, number of collisions, and out of tracks). MyoSuit [47] focused majorly on comfort and weight while maintaining its efficiency in aiding its users (ie, people with incomplete SCI, stroke, and multiple sclerosis or muscle dystrophy). Using elastomer springs and a *tendon driver unit*, MyoSuit was designed to act as an antigravity support during gait rehabilitation tasks. However, it was tested on only 1 patient with incomplete SCI, and so it is difficult to evaluate its efficiency and applicability for gait rehabilitation. The proposed EMC [51] focused on the simultaneous detection of multiple obstacles at different levels (in terms of height and distance) and floor status. EMC was designed using 6 ultrasonic sensors, a liquid detection sensor, a metal detection sensor, a wireless transceiver, and microcontroller circuits. Sensors were positioned on the stick to detect floor-level to head-level obstacles, as well as for leftward and rightward detection. EMC effectively provided navigation assistance, and the categorization or prioritization of detected information was better than with the white cane. However, more training time was suggested (even after a lengthy 20-hour training time) to properly ascertain its cognitive and perceptual load in comparison with similar devices.

Promising devices, such as EyeCane [52] and intelligent smart walker [49], had drawbacks as certain testing scenarios were not considered. EyeCane was tested only indoors, and the intelligent smart walker was tested using healthy patients who do not truly represent the typical users of the walker. The smart wheelchair that was designed to accommodate multiple control interfaces lacked a detailed evaluation of the performance and intelligence of the wheelchair for each interface. An example scenario is how the wheelchair would differentiate the user’s voice from an outlier when an alternative command option is in use. Therefore, there is a need for more detailed testing and evaluation before these technologies become usable and acceptable to their intended users.

#### CVI Systems

CVIs play an important role in the perception of mobility devices for autonomous navigation. CVI has been adopted in some technologies. For instance, See ColOr [63] was designed as a framework for the coupling of optical sensors in the context of range and color image registration. A sonic code was developed to map colors and depth into musical instruments. However, as it was the first of its kind, extensive training was required for the participants to master it, and testing was limited to certain scenarios (outdoor scenarios were not considered). A similar drawback was observed with patients with visual impairment [65]. It was designed to address the pervasiveness requirement (by integrating both an infrared light–based depth sensor and a stereo vision system together with an IMU device) as well as offer sensory substitution via sound feedback to patients with visual impairment. It was designed to work in any environment and illumination condition using sensor fusion techniques. The results seemed promising; however, detection or 3D representation of small objects or objects close to the ground needed a lot of improvement. In addition, only testing for indoor scenarios was conducted. iChair, was designed by Leaman et al [30], to accommodate a large range of impaired users by integrating multiple interfaces for control; however, no bug-free human trial has been documented. The same drawback was noted in the autonomous scooter [13], which was designed to be a cost-effective autonomous scooter that addressed the navigation and localization challenges in an unknown environment with a new hybrid far-field and near-field mapping solution.

The work toward autonomous navigation of mobility devices is ongoing and progressive but not without its challenges. This is because many stages make up the autonomous navigation system, and therefore, the overall performance can be hampered by just a small percentage error in one of its many stages. The first stage, the perception stage, is crucial to the performance of an autonomous navigation system as it has to do with the acquisition and processing of information. This stage, to a very large extent, determines the adaptability of the mobility device to the needs of the user. Different USIs are used to accommodate users with varying impairments; however, the ability to adequately adapt the mobility device is dependent on the quality of the information it receives. Many of the reviewed technologies applied different machine learning tools (classifiers and algorithms) to help process the acquired information. An example is the SVM classifier used by JPL BioSleeve in the studies by Assad et al [44] and Wolf et al [45] to classify gesture patterns. It was able to achieve an accuracy as high as 96%; however, as stated by Anguita et al [86], its accuracy was dependent on the chosen model, presence of noise, and data size. Drawbacks can be better tested by the comparison of similar classifiers to know which performs better for a particular technology, as was done by Wade et al [48].

In recent years, the idea of fusing data acquired from multiple sensors to improve confidence has been widely adopted because of the complementary properties exhibited by different sensors. Although this has proven to be promising, it does not come without its challenges [24,87]. This is majorly applied to CV. Examples include CV for patients with visual impairment [65] and the autonomous scooter [13], which used the fusion of 2 sensors for improved performance. To design CV for patients with visual impairment, a stereo red-green-blue camera (which is unreliable for depth estimation in the presence of poor illumination) was fused with a depth-of-field camera (which does not cope with bright light from the sun) in an attempt to improve the reconstructed 3D image output under any environmental condition. In the design of the autonomous scooter, long-range laser data were fused with that of a stereo vision camera to improve confidence under any environmental condition. Although fusion of data shows promising results, its efficiency is dependent on the accuracy of the applied fusing methods. An extensive literature review on fusion algorithms and the complementary properties of perception sensors and systems has been discussed by many researchers [24,69]. Some notable challenges in autonomous navigation and CV include improved accuracy and robustness in data fusion, trade-off between cost and performance, the self-localization problem, the detection of small or far-away objects in real time, training data set and increased testing scenarios, level of autonomy, and user training [23-25,60].

### Limitations and Future Directions

#### Overview

This study presents a comprehensive review of the recent literature on the adaptability of assistive mobility devices in terms of the acquired information. Discussions that present interesting facts and technical details regarding recent technologies have been reported. On the basis of the literature review, the following challenges and research directions are presented:

#### Improved Training Time and Avoidance of Cognitive Overload

Although the exact figure for the attention span of an average human being is extremely variable, research shows that the attention span of an average human being declines as the required concentration time increases. Therefore, it is widely accepted that keeping it simple is better. This is not different from the training time for users with some form of disability [88-91]. As highlighted in the *Brain-Computer Interface* section under *Discussion*, most of the reviewed prototypes showed that training time requires more attention. In addition, in the *Computer Vision Interfaces* section, the training time needed for machine learning algorithms varied depending on the training data set, which could affect the decision made in autonomously navigating assistive mobility devices [24]. More research could be conducted to improve the accuracy of BCI options with shorter training times and hybrid BCIs. This could be achieved with the help of machine learning techniques or algorithms that study user inputs and behaviors to accurately predict commands and help reduce the number of failed commands. Finally, a widely accepted standard for validating the training time for both machine learning algorithms and BCI in a USI could be developed. This will help researchers adequately compare results and monitor improvements concerning the adaptability of assistive mobility devices.

#### Accuracy

The data reveal that people who are adapted to using their wheelchairs have little to no tolerance for new functional errors. This situation is similar to that of every other assistive mobility device [92]. The highlighted technologies related to autonomous navigation (perception) and CV have shown that the data fusion technique has become increasingly accepted in improving accuracy. However, this also increases the complexity and robustness of information, thereby presenting challenges such as fusion, calibration, and classification accuracy [23-25,60]. Machine learning tools or algorithms used in processing this information also have varying strengths and weaknesses. Similar to the SVM classifier highlighted earlier, these tools and algorithms show varying accuracy depending on the selected model and the level of noise. Future research could be directed toward improving the accuracy of mobile robots in unfamiliar environments as this is mostly the case for assistive mobility devices.

#### IoMT Latency, Security and Privacy

The integration of IoMT frameworks with the highlighted technologies shows a lot of promise in improving the adaptability of assistive mobility devices to their users. With the IoMT technology option, data stored in the cloud can be analyzed and used for further research. The user’s progress (for gait rehabilitation) can also be monitored, and some level of assistive control can be done by the user’s stakeholders. However, with IoMT technology come network scalability, user privacy, and security problems [1,83,84]. Most reviewed papers acknowledged the packet loss problem when remotely controlling mobility devices via an IoMT framework and proposed various management systems to combat this problem; however, only simulation tests were carried out. The scalability of these frameworks can only be known when real-world testing is performed. Frameworks such as the robotic operating system [81] and the network-based rehabilitation system [79] may have major issues when implemented on a larger network scale. Further research could be conducted on management systems and algorithms developed to improve latency and compensate for packet loss. The developed frameworks should also indicate the number of devices that they could accommodate without any drop in performance. This could all be included in comprehensive system validation. Finally, a widely accepted standard for validating these systems or prototypes could be developed to help researchers compare results and documents on IoMT-based assistive mobility devices.

#### Performance Evaluation

In most of the reviewed papers, little attention was paid to real-world testing and comparing related prototypes to evaluate performance. For these technologies to be tagged fit for their intended users, their performance needs to be properly evaluated and tested under varying conditions. Proper evaluation would help examine some notable drawbacks, such as ease of use (without the need for any special training), cognitive overload (during human-machine communication), and the ease of wearing these technologies (in terms of weight while maintaining or improving their functionality) [15,20,21,31,43]. Some users of assistive mobility devices have comorbidities, such as mental health challenges because of aging or depression. If the training time or cognitive or perceptual load is high, the device will be quickly abandoned by its intended users. From the discussions, it has been shown that machine learning tools play a key role in the proper classification and processing of USI information as well as the decision-making of these mobility devices. These account for the ability of these devices to navigate autonomously with high accuracy. Future research could focus on the standardization of performance evaluation methods and the accepted testing conditions.

Another research direction is *the design of prototypes for clearly defined users.* As discussed in previous sections, specific USIs are most suitable for specific ailments. With the advent of many different USIs, there is a tendency to want to accommodate a wider range of users in a prototype design. When assistive mobility devices are tailored to specific users or ailments, there will be improved performance and accuracy in the adaptability of those devices to their specific users.

For a mobility device to be termed adaptable, it has to meet certain requirements such as the following:

1. *Intelligent perception*, that is, requires little or no effort to efficiently perceive its environment and take mobility decisions (such as obstacle avoidance and collision detection)
2. *Accurate self-localization* of user and device (user tracking).
3. *User-friendly*, that is, the movement speed and direction are controlled by the user subconsciously without the need for any special training; in addition, prompt and adequate control or feedback from and to the user are provided without cognitive overload, and communication with necessary stakeholders is easy and secure

These are needed for developed assistive mobility technologies to be easily commercialized and gain user acceptance (widespread adoption) [28,31]. These basic requirements reflect the need to evaluate the performance of mobility devices according to their major adaptability elements (ie, USIs, perception of adaptability—autonomous navigation—and IoMT framework).

### Conclusions

The research community has developed many promising technologies in the past decade, taking advantage of smart sensors, machine learning tools, and IoMT frameworks to offer mobility independence to impaired individuals. For users to benefit from these technologies, adaptability must be properly evaluated and considered from design to implementation. This study has successfully reviewed recent technologies of assistive mobility devices to identify their adaptability to users in terms of USI, autonomous navigation (perception stage), and connectivity. Tables have been presented to highlight the reviewed technology according to the major adaptability elements. Furthermore, the review presents some notable limitations, which have shown the need for improved cohesion to effectively adapt these technologies to their users. The findings discussed in the review show that for improved adaptability, more work needs to be done to reduce the training time and cognitive overload in the USIs to improve the fusion and classification accuracy; real-world scenario testing needs to be conducted and evaluated, and the trade-off between cost and performance needs to be considered in commercialization.

## Abbreviations

AR: autonomous robot
AV: autonomous vehicle
BCI: brain-computer interface
CV: computer vision
CVI: computer vision interface
EMC: electronic mobility cane
EMG: electromyography
IMU: inertial measurement unit
IoMT: internet of medical things
IoT: internet of things
MI: motor imagery
PRISMA: Preferred Reporting Items for Systematic Reviews and Meta-Analyses
PWC: powered wheelchair
SCI: spinal cord injury
SHS: smart health care system
SSD: sensory substitution device
SSSEP: steady-state somatosensory evoked potential
SSVEP: steady-state visual evoked potential
SVM: support vector machine
TDS: tongue drive system
TDS-iPhone-PWC: tongue drive system to smartphone (iPhone) electric-powered wheelchair
USI: user system interface
